# Development and pilot testing of a decision aid for drivers with dementia

**DOI:** 10.1186/1472-6947-14-19

**Published:** 2014-03-19

**Authors:** John Carmody, Jan Potter, Kate Lewis, Sanjay Bhargava, Victoria Traynor, Don Iverson

**Affiliations:** 1 Department of Neurology, Wollongong Hospital, Wollongong, NSW 2500, Australia; 2 Faculty of Science, Medicine and Health, University of Wollongong, Wollongong, NSW 2522, Australia; 3 Illawarra Health and Medical Research Institute (IHMRI), University of Wollongong, Wollongong, NSW 2522, Australia; 4 Department of Aged Care, Wollongong Hospital, Wollongong, NSW 2500, Australia

**Keywords:** Automobile, Decision aid, Decision making, Dementia, Driving, Patient education

## Abstract

**Background:**

An increasing number of older adults drive automobiles. Given that the prevalence of dementia is rising, it is necessary to address the issue of driving retirement. The purpose of this study is to evaluate how a self-administered decision aid contributed to decision making about driving retirement by individuals living with dementia. The primary outcome measure in this study was decisional conflict. Knowledge, decision, satisfaction with decision, booklet use and booklet acceptability were the secondary outcome measures.

**Methods:**

A mixed methods approach was adopted. Drivers with dementia were recruited from an Aged Care clinic and a Primary Care center in NSW, Australia. Telephone surveys were conducted before and after participants read the decision aid.

**Results:**

Twelve participants were recruited (mean age 75, SD 6.7). The primary outcome measure, decisional conflict, improved following use of the decision aid. Most participants felt that the decision aid: (i) was balanced; (ii) presented information well; and (iii) helped them decide about driving. In addition, mean knowledge scores improved after booklet use.

**Conclusions:**

This decision aid shows promise as an acceptable, useful and low-cost tool for drivers with dementia. A self-administered decision aid can be used to assist individuals with dementia decide about driving retirement. A randomized controlled trial is underway to evaluate the effectiveness of the tool.

## Background

The rising global prevalence of dementia represents an increasingly important medical, societal and economic issue. Alzheimer’s Disease International (ADI) and the World Health Organization (WHO) identified dementia as a public health priority [1]. Worldwide, there are more than 35.6 million people living with dementia [1]. By 2050 this figure is projected to rise to 115 million and the ADI and WHO have called for a more dementia friendly society [1]. To achieve this goal there needs to be improved planning and provision for individuals living with dementia [1,2].

Dementia is a condition characterized by impairment of memory and at least one other cognitive domain (e.g. executive function, language, praxis) which interfere with daily function and independence [3]. The incidence and prevalence of dementia increase with age [4]. Although Alzheimer’s disease is the most frequent cause of dementia, other neurological disorders can be responsible (e.g. vascular dementia, Lewy Body dementia, frontotemporal dementia). For many patients, symptoms begin insidiously and may pass unnoticed for some time [4]. As the condition progresses, the ability to drive safely is eventually lost [5]. Yet, many individuals continue to drive after receiving a diagnosis of dementia [6,7].

As our population is ageing, the number of older drivers is increasing [8-10]. Twenty years ago, 14% of all license holders in the United States were aged 65 years or more [11]; today it is 16.3% [12]. In the United Kingdom, 18.8% of the driving population is over 65 years [10]. This dependence by older individuals upon private cars is multifactorial [13,14]: (1) access to a car provides a sense of control, self-worth and independence [15,16]; (2) use of a car can enhance social interactions [17]; (3) alternative forms of transport are often lacking [18]; and (4) older drivers seldom plan for retirement from driving [18]. Furthermore, driving retirement is negatively associated with depression [19], difficulty accessing services [20] and nursing home placement [21].

The subject of driving and dementia raises a range of important ethical and medico-legal issues [15,16,22-24]. In essence, there is a need to balance road safety with the transport requirements of our ageing population [5,25-28]. Unfortunately, much of the literature relating to driving and dementia focuses upon safety rather than mobility [25]. Achieving the correct balance can prove elusive as, despite the existence of evidence-based clinical guidelines [29,30], many physicians simply do not raise the topic of driving retirement with individuals living with a dementia [31-33]. The need for such discussions is underscored by the fragility of older drivers and their elevated risk of injury in car crashes [29].

The majority of older drivers do not have dementia. However, given that increasing age is the leading risk factor for developing dementia [34], it is reasonable to expect more and more drivers with dementia on our roads. Thus, there is a pressing need to assist people with dementia in their decision making regarding retirement from driving. The overall purpose of this research project is to establish how a self-administered decision aid (DA) can assist drivers with dementia make decisions about driving retirement. The primary outcome measure was decisional conflict. The secondary outcome measures were knowledge, decision, satisfaction with the decision, booklet use and booklet acceptability.

Use of such a DA promotes a shift in focus away from assessment of fitness to drive. Rather, it emphasizes the need to facilitate planning for driving retirement. Such preparation for driving retirement has been likened to a ‘Ulysses contract’ [26,35] (Ulysses asked his crew to tie him to the ship’s mast on the condition that they ignored his pleas to be released when seduced by the song of the sirens [26]). It is anticipated that, by adopting a collaborative approach, individuals living with a dementia will be more likely to raise the subject of driving retirement with their family, carer or healthcare professional.

### Theoretical considerations

Decision making refers to the process of making choices between different courses of action or inaction; this process involves weighing up uncertain positive and negative outcomes, leading to decisional conflict [36,37]. DAs facilitate patient involvement in decisions about their healthcare leading to decisions which are informed and consistent with one’s values [38,39]. A recent Cochrane review [40] established that DAs: (1) improve knowledge; (2) reduce decisional conflict; (3) clarify expectations of possible benefits and harms; (4) lead to choices consistent with informed values; and (5) result in greater participation in decision making. Furthermore, DAs appear to have a positive effect on communication with health professionals despite a variable effect on actual choices [40]. Given that patients’ healthcare needs and preferences vary, it is appropriate to tailor communication strategies accordingly [41].

The Ottawa Decisional Support Framework (ODSF) [42] is a theoretical framework which is used to address the uncertainty or decisional conflict which may arise around healthcare choices. This framework consists of three components: (1) decisional needs; (2) decisional support; and (3) decisional quality. In line with this framework, the authors aimed to meet the decisional needs of drivers with dementia by providing them with adequate support so as to enhance the quality of their decision making process. The ODSF has been used to develop other dementia-related DAs: (1) respite service choices by carers of people with dementia [43]; and (2) feeding options in end-stage dementia [44].

## Methods

### DDDA development

The structure of this *driving with dementia decision aid* (DDDA) was informed by a wide range of resources: (1) the ODSF [42]; (2) the Ottawa Personal Decision Guide [45]; (3) the Australian National Health and Medical Research Council guide ‘How to prepare and present information for consumers of health services’ [46]; and (4) the International Patient Decision Aids Standards (IPDAS) collaboration guidelines [47].

The content of the DDDA was derived from a three-step approach. Firstly, relevant literature addressing driving and dementia was reviewed [24,27,32]. The attitudes of drivers aged over 55 towards existing driving and dementia resources were also sought. This served to clarify important deficiencies which apply to currently available resources for individuals planning to retire from driving [48]. Secondly, a development panel was formed which consisted of two clinicians and two senior academics. A draft DDDA was created and refined by the development panel using an iterative process. Thirdly, an expert review panel provided feedback on the draft DDDA. The panel comprised nine members from Australia (n = 7), Canada (n = 1), and the United Kingdom (n = 1), three of whom had experience in the development of DAs. Responses were sought around five categories: (1) layout; (2) reading ease; (3) length; (4) accuracy; and (5) relevance. The findings were used to modify the draft DDDA.

### DDDA presentation

The DDDA booklet (see Additional file 1) opens with a brief introduction which is followed by a guide on how to use the DA. Readers then progress through four key steps: (1) clarification of decision and values; (2) decisional needs and support; (3) considering the options; and (4) advising others of one’s decision. Information about the impact of dementia upon driving skills is included, and contact details for the Australian National Dementia Hotline are provided. To enhance reader engagement, detailed content (e.g. author affiliations, disclaimers, funding, references, scheduled updates) is provided at the end of the booklet.

In line with the recommendations of health communication experts [46,49,50], a range of strategies were used to enhance reader understanding of the content of the DDDA. Information was presented clearly (e.g. large font size, A4 sized pages) and concisely (e.g. 12 pages long, short sentences). A Flesch reading ease of 84.1 and a Flesch-Kincaid grade level of 3.8 were achieved suggesting that most 4^th^ grade students would be capable of reading the booklet. The pilot version of the DDDA fulfills 40 of 47 IPDAS collaboration quality criteria and is registered on-line with the Ottawa Hospital Research Institute decision aid library inventory [47,51]. The seven unmet quality criteria relate to the effectiveness of a DA and will be addressed in a randomized controlled trial.

### Pilot study

This pilot study involved a pre and post study design. Recruitment was undertaken over eight months in 2012. Ethical approval was provided by the regional Human Research and Ethics Committee and the local health district Research Governance Office. Potential participants were approached at two sites in regional New South Wales, Australia: (1) a university-affiliated tertiary hospital Aged Care dementia clinic; and (2) a community-based Primary Care center. Inclusion criteria consisted of: (1) a history of dementia (self-reported or clinically confirmed) regardless of duration or severity; (2) current driver; (3) ability to read English; and (4) ability to provide written consent to participate. Individuals who no longer drive were excluded. A convenience sampling technique was used to recruit participants; thus, individuals living with a dementia who were patients within these services were approached about possible participation. Potential participants were asked by their treating clinician if they were interested in learning more about a study on driving and dementia.

### Procedures

Individuals who expressed interest in becoming involved in this study were provided with a Participant Information Sheet (PIS) and a consent form. Signed consent forms were returned via reply-paid envelope. A research assistant telephoned each participant to complete a pre-booklet survey. The pilot version DDDA was then mailed to participants. One week later, a post-booklet survey was conducted thus affording participants adequate time to reflect upon contents of the booklet.

### Measures

Participant demographics, knowledge, decision (about driving retirement) and decisional conflict were recorded during the pre-booklet survey. Except for the demographic details, the post-booklet survey assessed the same measures in addition to booklet use, booklet acceptability, and satisfaction with decision. In addition, open-ended questions were included at the end of the post-booklet survey to assess the acceptability of the DA by participants.

The primary outcome measure, decisional conflict, was measured with a low-literacy decisional conflict scale [52] previously used in other DA studies [53]. This scale measures personal perceptions of: (i) uncertainty in choosing options; and (ii) modifiable factors contributing to uncertainty [52]. The secondary outcome measures (knowledge, decision, satisfaction with decision, booklet use and booklet acceptability) were assessed using existing tools. Dementia knowledge was measured using a 10-item survey based on the Ottawa knowledge questionnaire template [54]. The content of the dementia knowledge quiz was informed by reviewing the available literature relating to dementia and driving. Participants’ decision regarding driving was recorded as: (1) stop driving now; (2) drive less; (3) stop driving later; (4) unsure; or (5) other. Satisfaction with decision was measured using a validated satisfaction with decision scale [55]. Booklet use was reviewed by establishing the length of time required to read the booklet, and whether assistance was required by the participant to use it. Booklet acceptability was measured using an 8-item survey adapted from the Ottawa acceptability tool [56]: this component of the post-booklet survey was used to seek feedback from participants on how the booklet could be improved. Descriptive statistical analysis was performed using SPSS version 21 (IBM, Chicago, USA).

## Results

Twelve participants completed the pre and post-booklet surveys: nine males and three females (see Table 1). The mean age was 75.4 (range 66–88, SD 6.7). The living arrangements of participants included living at home with a spouse/partner (*n* = 9), living alone (*n* = 1) and living in a nursing home (*n* = 2). The highest level of education among participants was: primary school (*n* = 3); high school (*n* = 3); certificate/diploma (*n* = 4); undergraduate degree (*n* = 1); and post-graduate degree (*n* = 1). All participants were either unemployed or retired. The mean duration of driving experience was 54.4 years (range 40–69, SD 7.9). One participant was instructed by a doctor to stop driving two weeks prior to entering the study but was up until that time, still driving and doing so daily. The remaining participants (*n* = 11) were active drivers: 2–6 times per week (*n* = 3); once a day (*n* = 4); and more than once a day (*n* = 4). A mean booklet reading time of 30 minutes was reported (range 5–60, SD 20.7). All but two participants (*n* = 10) read the booklet without assistance.

**Table 1:** Demographic characteristics of study participants

**Variable**	**Participants (*N *** **(%))**
Age (mean)	75.4 years
Gender	9 males/3 females
Living arrangements	
With spouse/partner at home	9 (75%)
With other family at home	-
Alone at home	1 (8.3%)
Hostel	-
Nursing home	2 (16.6%)
Other	-
Highest level of education	
Primary school	3 (25%)
High school	3 (25%)
Certificate/diploma	4 (33.3%)
Undergraduate degree	1 (8.3%)
Postgraduate degree	1 (8.3%)
Employment status	
Employed	-
Unemployed/retired	12 (100%)
Volunteer worker	-
Length of driving experience (mean)	54.4 years
Driving frequency	
Less than once a week	-
Once a week	-
2-6 times per week	3 (25%)
Once a day	4 (33.3%)
More than once a day	4 (33.3%)

The mean knowledge score was 5.3 pre-booklet (SD 2.4); this rose to 5.8 post-booklet (SD 2.6) (maximum possible score = 10). When asked which driving option was preferred, participants chose: stop driving now (*n* = 10 pre-booklet, *n* = 7 post-booklet); drive less (*n* = 0 pre-booklet, *n* = 1 post-booklet); stop driving later (*n* = 1 pre-booklet, *n* = 1 post-booklet); and unsure (*n* = 1 pre-booklet, *n* = 3 post-booklet). The low-literacy decisional conflict scale results range from zero to 100 (a high score indicates high decisional conflict) [52]. A mean score of 22.5 was recorded pre-booklet (range 0–60, SD 17.1); this fell to 7.5 post-booklet (range 0–30, SD 9.7). Post-booklet satisfaction with decisions about driving retirement was high (mean 4.68/5, range 4.16-5, SD 0.3).

All participants found the length and information content of the DDDA to be ‘just right’ (see Table 2). A large majority described the booklet as balanced (83.3%) with information presented in a ‘good’ or ‘excellent’ manner (91.6%). Most participants (91.6%) felt that the DDDA helped them decide about driving and all would recommend the booklet to others. Qualitative feedback regarding the booklet was favorable (see Table 3).

**Table 2:** Decision aid acceptability

**Variable**	**Participants (*N *** **(%))**
Information presentation	
Poor	-
Fair	1 (8.3%)
Good	3 (25%)
Excellent	8 (66.6%)
Booklet length	
Too long	-
Too short	-
Just right	12 (100%)
Was there enough information to decide about driving?	
Too much information	-
Too little information	-
Just right	12 (100%)
Was the booklet balanced?	
Slanted against driving	1 (8.3%)
Slanted in favor of driving	1 (8.3%)
Balanced	10 (83.3%)
Was the booklet useful in helping decide about driving?	
Yes	11 (91.6%)
No	1 (8.3%)
Would you recommend the booklet to others?	
Yes	12 (100%)
No	-

**Table 3:** Qualitative feedback from participants and family

**Question**	**Responses**
**Was the booklet useful in helping decide about driving?**	• Found it very useful.
	• Did not feel it was relevant for me.
	• Interesting – made him [husband] think about the issue. Had not really considered it before.
	• Very helpful. Used it to have a roundtable discussion with grown children and husband.
**What did you like about the booklet?**	• Reasonably fair and easy to read.
	• Well set out, clearly organized, easy to understand.
	• The checklists were helpful.
	• A lot of good information. It included things that people need to know. Enjoyed filling check boxes.
	• Very easy to navigate. The options in the checklists are very comprehensive. All steps are very clear.
	• The booklet brought home some things that we had already been thinking about, and helped to put them into practice. It has made us change the way we do things. It is brief, to the point.
	• The content is very relevant to others, not just dementia. Good to use as a tool to start conversation with others.
**How do you think we could improve the booklet?**	• No, it covers everything well.
	• Have more people review it.
	• Be more specific when referring to doctor – do you mean General Practitioner?

## Discussion

The purpose of this research was to establish if a self-administered DA can assist drivers with dementia make decisions about driving retirement. This pilot study provided an opportunity for individuals with dementia, who are often excluded from medical research, to express their views about the decision to retire from driving [57]. Overly restrictive study protocols often preclude the recruitment of older participants [58], and particularly people with cognitive impairment or multiple co-morbidities. Thus, individuals with dementia can be denied access to new interventions or therapies. This study helped to redress this imbalance through the development and preliminary evaluation of a DA for drivers with dementia.

Most participants completed the booklet without assistance, requiring an average reading time of 30 minutes. A concerted effort was made during the development phase to ensure the study booklet was clear, concise and sensitive to the needs (e.g. cognitive requirements) of individuals with dementia. As reported in the development of a low literacy DA elsewhere [59], simple strategies were employed to improve the readability of the DDDA and reduce the cognitive effort required [50] by using: (1) large font size; (2) active voice; (3) short sentences; and (4) simple diagrams. Consequently, a low Flesch-Kincaid reading grade level of 3.8 was achieved.

The IPDAS collaboration criteria [47] serve as a validated measure of DA quality, as well as a useful guide in the development of new DAs. The DDDA rated highly in two of three quality domains: (1) content 20/20; (2) development process 20/20; and (3) effectiveness 0/7. The final version of the DDDA booklet will be forwarded to the IPDAS instrument assessment team in Cardiff, United Kingdom [59] for an objective assessment against IPDAS quality criteria. This will serve two important functions: (1) confirm that the DDDA has undergone comprehensive and rigorous development; and (2) provide assurance that it satisfies internationally agreed standards of quality.

It is widely acknowledged that the recruitment of individuals with dementia is fraught with challenges [60,61]. Accordingly, a limitation of this pilot study is its low sample size. An additional limitation is the absence of delayed follow-up data (e.g. six month follow-up survey). Notwithstanding these limitations, encouraging improvements in participant knowledge and decisional conflict were observed following use of the DDDA. In addition, booklet acceptability was high and qualitative feedback from participants was favorable. In view of these preliminary findings, a randomized controlled trial (RCT) has been initiated to better understand the clinical impact of the DDDA (ACTRN 12613000174785). A potential limitation of this pilot study is the nature of the literature review which informed the development of the DDDA. A systematic review was not undertaken: (1) to avoid undue replication of existing reviews; (2) as a low yield of additional relevant studies was anticipated; (3) as it was unlikely to alter the DDDA development; and (4) as it was unlikely to alter the methods, results or outcomes of this pilot study.

## Conclusion

Discussion with individuals with dementia about driving retirement often represents a challenging clinical encounter for health professionals [24,27]. A Pyrrhic victory may ensue whereby individuals with dementia are instructed to cease driving yet they neither heed their clinician’s advice nor return for medical review. Thus, there exists a clear need to facilitate conversations related to early retirement from driving. Ideally, such discussions would occur shortly after a diagnosis is reached. This pilot study demonstrates how a multi-faceted approach (i.e. development panel, review panel and field testing) resulted in the creation of a feasible and acceptable DA for individuals with dementia. This DDDA provides a simple and balanced outline of the benefits and risks of driving. It facilitates clarification of values, promotes planning for retirement from driving and encourages the reader to speak with their doctor. The DA resource was developed in line with the IPDAS collaboration guidelines [47] and pilot tested by drivers with dementia. However, further research is required to evaluate the impact of this DA in the target group. Accordingly, a randomized controlled trial of drivers with dementia is currently underway.

There is a need for a comprehensive and inclusive approach to older drivers with cognitive impairment [24,27,33,62]. This study describes an intervention which contributes towards the achievement of an important goal: enhancing patients’ quality of life while simultaneously maintaining personal and public safety [62]. It is intended that, ultimately, the DDDA will be made freely available to patients, carers and clinicians by providing copies to (1) local, state and national healthcare authorities, (2) national road safety organizations, and (3) relevant consumer support groups. The booklet is designed to facilitate discussion about a frequently neglected issue: driving retirement by individuals with dementia. Although the focus of this study was on drivers with dementia, the methods used should guide future DA development (e.g. driving and epilepsy, driving and sleep apnea, dementia and management of finances).

## Abbreviations

ADI: Alzheimer’s Disease International; DA: Decision aid; DDDA: Driving with dementia decision aid; IPDAS: International patient decision aids standards; PIS: Participant information sheets; SD: Standard deviation; SPSS: Statistical package for the social sciences; WHO: World Health Organization.

## Competing interests

The authors declare that they have no competing interests.

## Authors’ contributions

JC, JP, KL, SB, VT and DI contributed to the concept and design of the study. JC secured ethical approval. JP, KL and SB contributed to data collection. JC prepared the manuscript which has been reviewed by the other authors. All authors have approved the final manuscript.

## Pre-publication history

The pre-publication history for this paper can be accessed here:

http://www.biomedcentral.com/1472-6947/14/19/prepub

## Supplementary Material

Additional file 1 Driving with dementia decision aid. Click here for file
