# Supplementary material for: Development and pilot testing of a decision aid for drivers with dementia
Source: BMC Med Inform Decis Mak. 2014 Mar 19;14:19. doi: 10.1186/1472-6947-14-19 (PMC3999924; doi:10.1186/1472-6947-14-19)
Supplement: Additional file 1 — Driving with dementia decision aid. [file 1472-6947-14-19-S1.pdf]

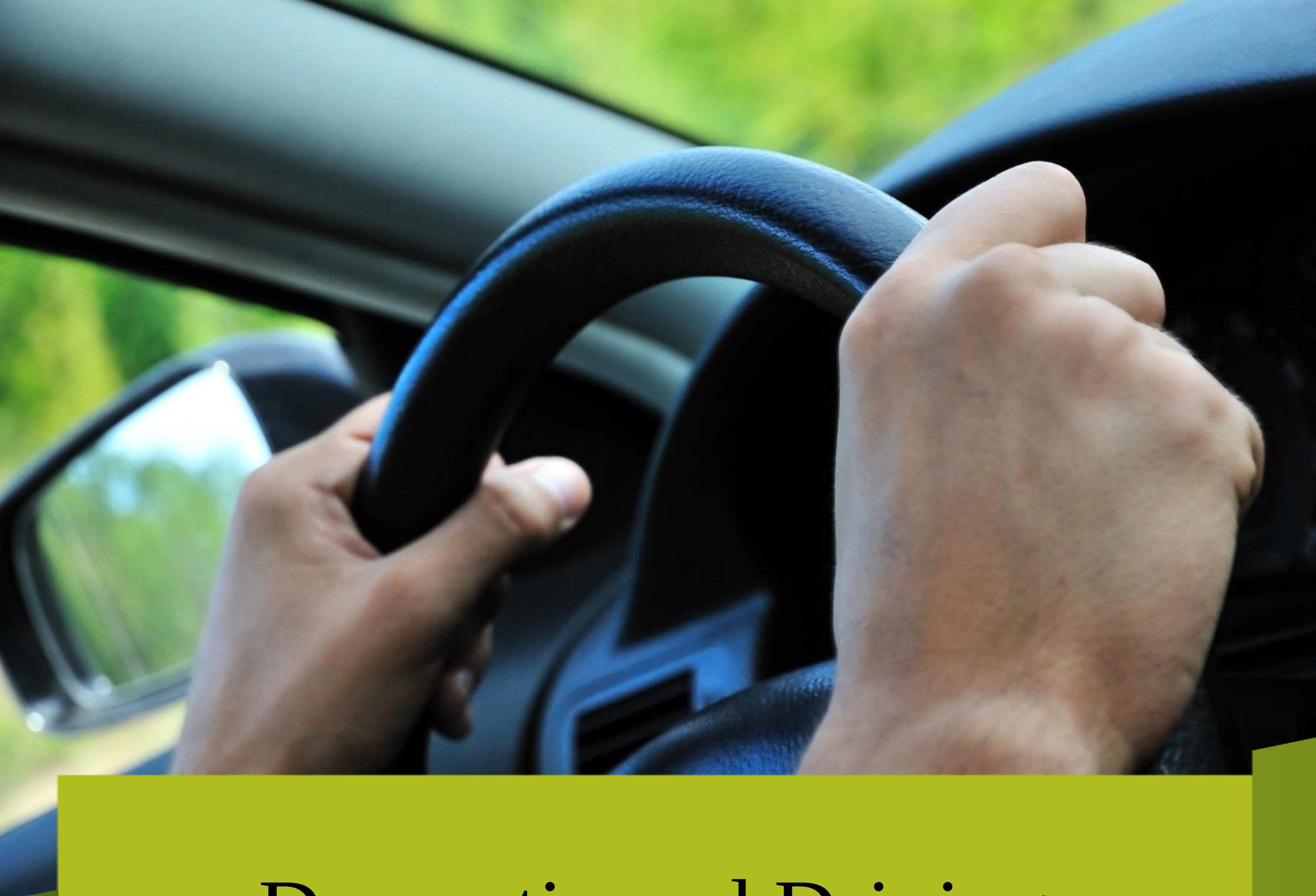

Dementia and Driving: a decision aid

Dr John Carmody FRACP

A/Prof Victoria Traynor PhD

Prof Don Iverson PhD

Ms Catherine Andrew MSc

UNIVERSITY OF WOLLONGONG

CONTENTS

Introduction and instructions Page 2

Step 1: **To help clarify your decision** Page 3

Step 2: **What do you need to make your decision?** Page 6

Step 3: **Weighing your options** Page 7

Step 4: **What next?** Page 10

References Page 13

Miscellaneous Page 14

DRAFT

INTRODUCTION

A diagnosis of dementia can come as quite a shock. It is accompanied by a variety of changes to one's lifestyle and needs. This booklet may be used by any driver who has dementia.

Most older adults have driven safely and remained accident-free for many years. Unfortunately, dementia can silently disrupt driving skills. At times, family members notice unsafe driving behaviour before you do. For a variety of reasons, the issue of driving safety is often not raised by doctors, nurses, family or friends.

The aim of this booklet is to assist you in deciding when to stop driving after receiving a diagnosis of dementia. It is hoped that early planning for retirement from driving will avoid the need to stop suddenly in the future.

DRAFT

INSTRUCTIONS

- Please read this booklet from beginning to end
- You will be guided through 4 steps
- Use a pencil to answer questions
- ■ Tick these green boxes if you agree
- Please write your answers on the dotted lines _ _ _ _ _ _ _ _ _ _ _
- You may reuse this booklet as often as you wish

STEP 1: TO HELP CLARIFY YOUR DECISION

What is dementia?

Usually, dementia is a condition which gradually disrupts one's memory, speech, concentration, judgement and ability to plan. The most common forms of dementia are Alzheimer's disease, vascular dementia, Lewy body disease and Frontotemporal dementia. About 6% of people over the age of 65 are affected. Dementia is associated with many other conditions and medication use. Some forms of dementia increase your risk of seizures and strokes.

Can dementia affect your driving skills?

Yes. Eventually, most people with dementia become unsafe to drive.

Driving is a complex task. In order to drive safely, you rely upon a variety of skills such as judgement, memory, attention and the ability to assess your surroundings. Unfortunately, people with dementia often lose these important skills. For many, this happens without you realising it. Over time, your risk of becoming lost or having a car accident rises significantly.

What are your options?

- Continue driving with no change
- Stop driving now
- Drive less
- Stop driving later

DRAFT

STEP 1: TO HELP CLARIFY YOUR DECISION

How far along are you with making a decision about driving?

- I have not yet thought about my options
- I am thinking about my options
- I have almost made my decision
- I have already made my decision

How will you cope without your car?

People with dementia wish to drive for a number of reasons: the pleasure of driving; independence; shopping; work; visiting family or friends; going to appointments; and travelling. Many are afraid to discuss driving safety for fear of losing their licence.

Fortunately, many helpful alternative forms of transport exist:

- Family or friends
- Public transport (e.g. bus, train, ferry)
- Taxi (subsidies are available for some)
- Relocating to more central location (e.g. moving to live closer to a town centre)

Many people underestimate how much money they could save by retiring from driving. One no longer needs to pay for annual registration, car insurance, maintenance checks, repairs and ever increasing fuel costs.

DRAFT

STEP 1: TO HELP CLARIFY YOUR DECISION

Have you noticed any warning signs of unsafe driving?

Driving errors are a sign that your driving skills may no longer be adequate. Have you experienced any of the following warning signs while driving?

- Find changing lanes difficult to do
- Find roundabouts difficult to use
- Drive very slowly
- Confuse left and right
- Become lost on short trips
- Drive on the wrong side of the road
- Notice damage to my car that I cannot explain
- Have car accidents

How often do you experience any of these warning signs?

- Never
- Rarely
- Sometimes
- Often

DRAFT

STEP 2: WHAT DO YOU NEED TO MAKE YOUR DECISION?

Support

- Do I have enough help or advice from others to make a choice? Yes ☐ No ☐
- Am I being forced by others to make a choice? Yes ☐ No ☐

Knowledge

- Do I know my options? Yes ☐ No ☐
- Do I know the benefits and risks of each option? Yes ☐ No ☐

Values

- Am I clear about which benefits and risks matter most to me? Yes ☐ No ☐

Certainty

- Do I feel sure about the best choice for me? Yes ☐ No ☐

DRAFT

STEP 3: WEIGHING YOUR OPTIONS

Support

- Who else is involved with my decisions about driving? _ _ _ _ _ _ _ _ _ _ _
- What option do they prefer? _ _ _ _ _ _ _ _ _ _ _ _ _ _

  Options = No change / Stop driving now / Drive less / Stop driving later

- Is this person pressuring me? ■ Yes ■ No
- Can this person help me? ■ Yes ■ No
- What do I prefer? ■ I prefer to decide for myself

  ■ I prefer to let someone else decide

  ■ I prefer to share the decision with others

DRAFT

Knowledge

Most experts would agree that drivers with dementia are at a higher risk of getting lost or having a car accident. Some researchers have found the risk of accident to be 2.5 to 10 times higher than other people of the same age. This is a difficult topic to research and your exact risk is uncertain.

What are the benefits of driving?

- Independence
- Pleasure of driving
- Visiting family & friends
- Going to appointments
- Travel

What are the risks of driving?

- Becoming lost
- Car accident
- Personal injury
- Injury to others
- Death

STEP 3: WEIGHING YOUR OPTIONS

Knowledge

Does your risk change over time?

Yes, it does. Your driving skills will worsen over time. Also, your risk of serious injury or death following a car crash rises as you get older. This bar chart shows the number of healthy drivers who are injured or killed in car crashes according to age. However, drivers with dementia are estimated to be at an even higher risk (up to 10 times greater).

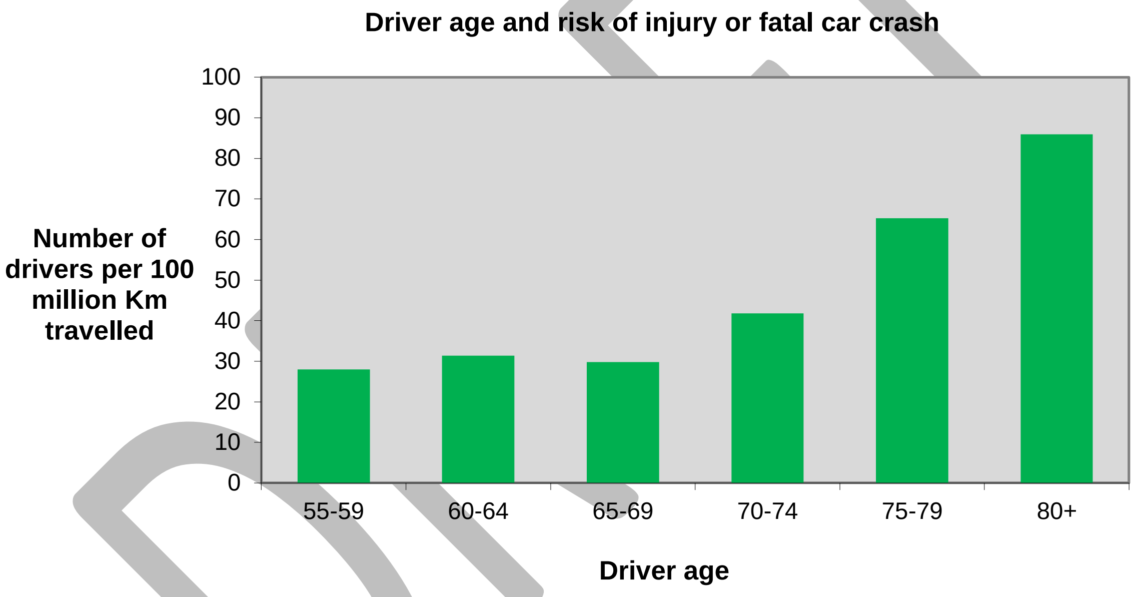

What are the guidelines in Australia?

The Australian and New Zealand Society of Geriatric Medicine says:

- Some people with mild dementia may be safe to continue driving for a limited period of time

The Austroads national guidelines state that:

- People with dementia may be eligible to hold a conditional driver licence
- Medical review of ability to drive is necessary every 12 months

STEP 3: WEIGHING YOUR OPTIONS

Values

What is the most important reason for you to continue driving?

- Independence
- Pleasure of driving
- Visiting family
- Visiting friends
- Going to appointments
- Travelling to new places
- Shopping
- Poor access to public transport
- Other _ _ _ _ _ _ _ _ _ _ _ _ _ _

What is the most important reason for you to stop driving?

- Risk of getting lost
- Risk of a car crash
- Risk of injury to me
- Risk of injury to others
- Crash leading to death
- Worried family
- Worried friends
- Other _ _ _ _ _ _ _ _ _ _ _ _ _ _

Of all the items on this page – which is the single most important item for you?

- _ _ _ _ _ _ _ _ _ _ _ _ _ _

DRAFT

STEP 4: WHAT NEXT?

Support

If you feel you do not have enough support to make a decision then you can seek additional support from others. Who do you prefer to speak to? You can use this later to remind you.

- My doctor (general practitioner or specialist)
- My family
- My friends
- National Dementia Hotline 1800 100 500
- Alzheimer's Australia counsellor 1800 100 500

Knowledge

If you do not have enough information to make a decision then you can ask for more facts from others. Where would you prefer to go to?

- My doctor (general practitioner or specialist)
- My local library
- National Dementia Hotline 1800 100 500
- Alzheimer's Australia website www.fightdementia.org.au
- Austroads website www.austroads.com.au

DRAFT

STEP 4: WHAT NEXT?

Values

Here are a few tips on how to clarify the values that mean the most to you. You may choose as many of these options as you wish:

- Talk to others who have made the decision
- Discuss with others what matters most to me
- Consider joining a local support group
    - details available from the National Dementia Hotline 1800 100 500

DRAFT

Questions

What questions need answering to help you decide? You may wish to show these to other people such as your doctor. Discussing this booklet with family and friends is often helpful.

1. _

2. _

3. _

STEP 4: WHAT NEXT?

Through this booklet you have now considered

- How dementia affects driving skills
- Your options regarding driving
- What you have noticed about your own driving
- What supports you have
- The reasons for and against driving
- Where to go for more information or support

Decision

Having read this booklet, have you reached a decision about driving? What have you decided?

- I will continue driving with no change
- I will stop driving now
- I will drive less
- I will stop driving later
- I am unsure

The Next Step

Please arrange an appointment to meet with your doctor to discuss this further. You may find it helpful to bring this booklet with you. Please reuse this booklet as often as you wish.

DRAFT

REFERENCES

Alzheimers Australia 2005, *About you driving: information for people with dementia*. www.fightdementia.org.au/common/files/NAT/20050700_Nat_HS_8.4Driving.pdf

American Psychiatric Association Diagnostic and Statistical Manual 1994, 4th ed. Washington DC: APA Press.

Australian and New Zealand Society for Geriatric Medicine 2009, *Position statement number 11: Driving and dementia*. www.anzsgm.org/posstate.asp

Austroads. *Assessing fitness to drive for commercial and private vehicle drivers: medical standard for licensing and clinical management guidelines*. 4th ed. Sydney: Austroads; 2012.

Breen, DA, Breen, DP, Moore, JW, Breen, PA and O'Neill, D 2007, 'Driving and dementia', *British Medical Journal*, vol. 334, pp. 1365-1369.

Carr, DB, Duchek, J and Morris, JC 2000, 'Characteristics of motor vehicle crashes of drivers with dementia of the Alzheimer's type', *Journal of the American Geriatric Society,* vol. 48, pp. 18–22.

Dubinsky, RM, Stein, AC and Lyons, K 2005, 'Practice parameter: risk of driving and Alzheimer's disease (an evidence-based review)', *Neurology,* vol. 54, pp. 2205-2211.

Man-Son-Hing, M, Marshall, SC, Molnar, FJ and Wilson, KG 2007, 'Systematic review of driving risk and the efficacy of compensatory strategies in persons with dementia', *Journal of the American Geriatric Society*, vol. 55, pp. 878–884, 2007.

Martin, AJ, Marottoli, R and O'Neill, D 2009, 'Driving assessment for maintaining mobility and safety in drivers with dementia', *Cochrane Database of Systematic Reviews*, issue 1.

Ministry of Transport 2011, *New Zealand household travel survey 2006-2010. Risk on the road: drivers and their passengers V2.0*, Wellington, New Zealand. www.transport.govt.nz/research/Documents/Risk-2011-Drivers-and-passengers.pdf

Thal, LJ, Grundman, M and Klauber, MR 1988, 'Dementia: characteristics of a referral population and factors associated with progression', *Neurology*, vol. 38, pp. 1083-1090.

Uc, EY, Rizzo, M, Anderson, SW, Shi, Q and Dawson, JD 2004, 'Driver route following and safety errors in early Alzheimer disease', *Neurology,* vol. 63, pp. 832–837.

Van der Flier, WM and Scheltens, P 2005, 'Epidemiology and risk factors of dementia', *Journal of Neurology, Neurosurgery and Psychiatry*, vol. 76, pp. 2-7.

Zuin, D, Ortiz, H, Boromei, D and Lopez, OL 2002, 'Motor vehicle crashes and abnormal driving behaviours in patients with dementia in Mendoza, Argentina', *European Journal of Neurology,* vol. 9, pp. 29-34.

DRAFT

MISCELLANEOUS

CONFLICT OF INTEREST

Financial support was provided by the (i) Wollongong Hospital, and (ii) NSW Roads and Maritime Services (RMS). Neither the authors nor their affiliated organisations stand to gain financially from the use of this booklet.

ADAPTED FROM

The Ottawa Personal Decision Guide © 2006, O'Connor, Jacobsen & Stacey. Ottawa Hospital Research Institute (OHRI), Ottawa, Canada.

DISCLAIMER

The aim of this booklet is not to replace the advice of your doctor or health professional. All efforts were taken to ensure the content of this booklet was accurate at the time of publication. This version (number 2, 2013) will be updated in 2 years (2015).

ACKNOWLEDGEMENTS

The authors wish to acknowledge the work of Prof Annette O' Connor and Prof Dawn Stacey (The Patient Decision Aids Research Group, OHRI, Canada). We wish to thank the reviewers of this booklet: A/Prof Guy Bashford; Dr Vida Bliokas; Prof Andrew Bonney; Ms Jenny Davies; Prof Glyn Elwyn's Decision Laboratory (Cardiff University, Wales); Ms Robyn Faine (Alzheimer's Australia NSW); Ms Kate Lewis; Prof Elena Marchetti; Prof Jan Potter; Mr Robert Reynolds (RMS, NSW); Mr Anton Saarimaki (OHRI, Canada); and Dr Christine Stirling. We would also to thank the patients, carers and family members who contributed to the creation of this decision aid. Without their kind assistance, this work would not have been possible. Lastly, the invaluable support of Mr Robert Reynolds, Roads and Maritime Services, Wollongong, NSW is gratefully acknowledged.

DRAFT

Dementia and Driving: a decision aid

DRAFT

AUTHORS

Dr John Carmody
MB BCh, MRCPI, FRACP
Staff Specialist Neurologist, Wollongong Hospital
Clinical Associate Professor, University of Wollongong

A/Prof Victoria Traynor
BSc (Hons), RGN, PGCHE, ILM, PhD
Associate Professor, University of Wollongong
Associate Director, NSW/ACT Dementia Training Study Centre

Prof Don Iverson
BSc, MSc, PhD
Executive Dean, Faculty of Science, Medicine and Health

Ms Catherine Andrew
BAppSc, MSc
Occupational Therapist
Injury management coordinator, University of Wollongong

NSW GOVERNMENT Health Illawarra Shoalhaven Local Health District

UNIVERSITY OF WOLLONGONG
